# The Beat

**Published:** 2009-02

**Authors:** Erin E. Dooley

## Post-Tsunami Coral Comeback

In December 2004, coral populations were devastated by the deadly tsunami that swept across the Indian Ocean. A new survey by the World Conservation Society found high densities of baby corals in 60 tsunami-ravaged locations in Aceh, Indonesia. The coral comeback is being linked to natural colonization by more resilient coral species along with a decline in drastic fishing practices such as the use of dynamite. In addition, transplanting baby corals from healthy wild coral has proved to be a more effective strategy than direct seeding for restocking coral-depleted areas.

**Figure:**
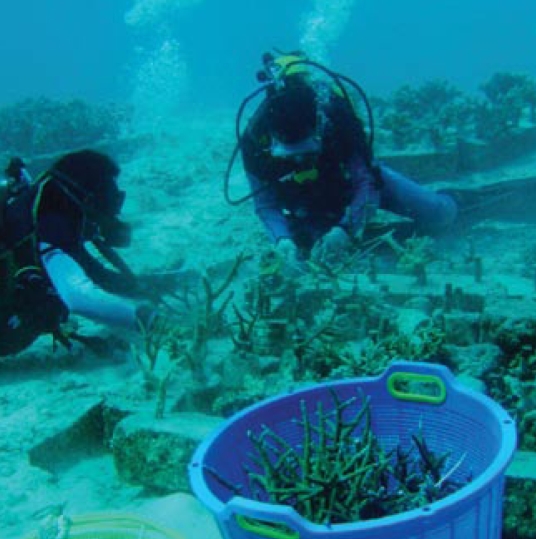
Divers transplant corals off Aceh, Indonesia.

## USDA Announces New Ecosystem Office

On 29 December 2008, Agriculture Secretary Ed Schafer announced the U.S. Department of Agriculture will, as directed by the 2008 Farm Bill, form a new Office of Ecosystem Services and Markets and create a federal Conservation and Land Management Environmental Services Board. These entities will assist the department in guiding the implementation of market-based approaches to conservation, such as compensating farmers, ranchers, and forest landowners for providing wildlife habitat, carbon storage, and scenic landscapes. The first service to be examined by the new office will be carbon sequestration.

## Australia Says No Endosulfan Ban

Despite concerns by domestic and international groups about the endocrine-disrupting effects and persistence in the environment of endosulfan, Australia will continue to allow the restricted use of this insecticide. This announcement was made in January 2009 by the Australian Pesticides and Veterinary Medicines Authority. In December 2008 Australia’s closest neighbor, New Zealand, joined 54 other countries in banning endosulfan. The Stockholm Convention on Persistent Organic Pollutants will determine in October 2009 whether to do a final assessment on endosulfan, which could result in a global ban by 2011.

## CPSC Revises Safety Act

In 2008, the Consumer Product Safety Improvement Act was enacted to bolster efforts to protect children from lead and phthalates in products such as toys and clothing. In response to concerns expressed by thrift store owners and purveyors of handmade toys, the Consumer Product Safety Commission voted in January 2009 to refine the act—for example, by relieving thrift shops of the need to test used goods for lead and phthalates. However, resellers and parents alike should avoid selling or buying used products that are likely to contain lead or phthalates.

**Figure:**
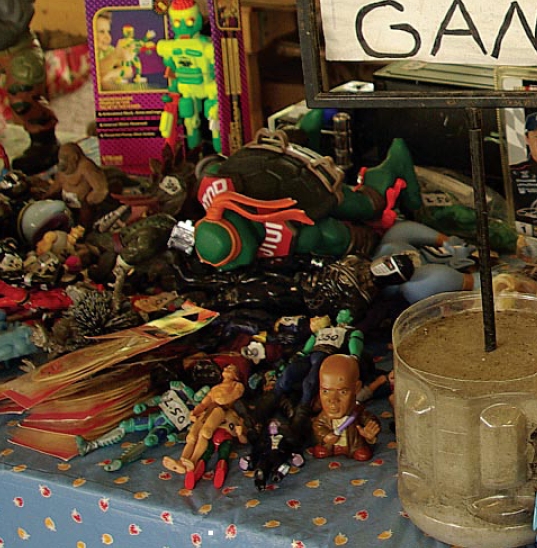
Secondhand toy sales will be governed by good sense, not legislation.

## New Tests Lower Lab Animal Needs

The U.S. Interagency Coordinating Committee on the Validation of Alternative Methods (ICCVAM) has endorsed two standardized, oral, cell-based tests to help companies determine the potential hazards of chemicals. The new methods measure cell death and can determine the first dose to test in animals. Acute oral toxicity tests are currently the most commonly used toxicity tests used in animals worldwide, and the new tests could greatly reduce the numbers of lab animals needed for such tests. ICCVAM is seeking incorporation of the test recommendations in guidance to be issued by the Organisation for Economic Co-operation and Development (OECD), which will go to OECD’s 30 member countries.

## Lights Out for Low-Efficiency Bulbs

In a move that could dramatically curb carbon emissions and European household energy costs, the European Union (EU) is now moving forward with formal legislation to ban low-efficiency light bulbs by 2012. The first phase of the legislation will begin in September 2009, when bulbs with a light output of 100 watts or more must achieve at least a C-class efficiency rating—a requirement that incandescent bulbs of this wattage will be unable to meet. The regulations will continue to be implemented in stages until all classes of bulbs have at least a C-class rating. According to an EU technical briefing on the new legislation, lighting can represent up to one-fifth of a household’s electricity consumption.

**Figure:**
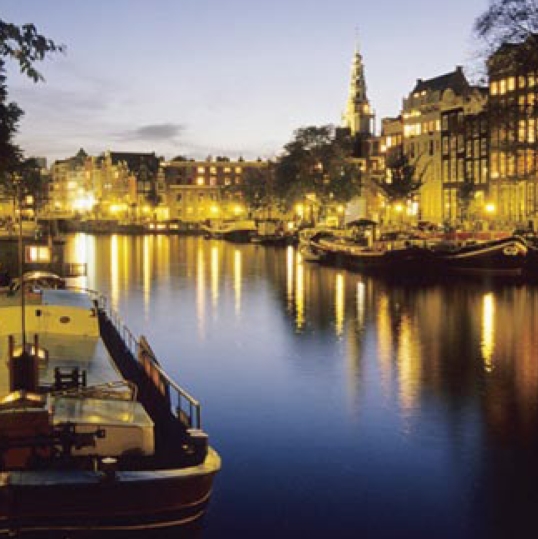
Amsterdam, Netherlands. European lighting will go high-efficiency by 2012.

